# Compositional Quality and Potential Gastrointestinal Behavior of Probiotic Products Commercialized in Italy

**DOI:** 10.3389/fmed.2018.00059

**Published:** 2018-03-07

**Authors:** Alessandra Vecchione, Francesco Celandroni, Diletta Mazzantini, Sonia Senesi, Antonella Lupetti, Emilia Ghelardi

**Affiliations:** ^1^Department of Translational Research and New Technologies in Medicine and Surgery, University of Pisa, Pisa, Italy; ^2^Department of Biology, University of Pisa, Pisa, Italy; ^3^Research Center Nutraceuticals and Food for Health-Nutrafood, University of Pisa, Pisa, Italy

**Keywords:** probiotics, microbial identification, MALDI-TOF, gastric juice, intestinal fluid, acid resistance, bile tolerance

## Abstract

Recent guidelines indicate that oral probiotics, living microorganisms able to confer a health benefit on the host, should be safe for human consumption, when administered in a sufficient amount, and resist acid and bile to exert their beneficial effects (e.g., metabolic, immunomodulatory, anti-inflammatory, competitive). This study evaluated quantitative and qualitative aspects and the viability in simulated gastric and intestinal juices of commercial probiotic formulations available in Italy. Plate counting and MALDI-TOF mass spectrometry were used to enumerate and identify the contained organisms. *In vitro* studies with two artificial gastric juices and pancreatin–bile salt solution were performed to gain information on the gastric tolerance and bile resistance of the probiotic formulations. Most preparations satisfied the requirements for probiotics and no contaminants were found. Acid resistance and viability in bile were extremely variable depending on the composition of the formulations in terms of contained species and strains. In conclusion, this study indicates good microbiological quality but striking differences in the behavior in the presence of acids and bile for probiotic formulations marketed in Italy.

## Introduction

Probiotics are defined as live microorganisms that, when administered in adequate amounts, confer health benefits to the host (1). The most commonly used oral probiotic formulations contain lactobacilli and bifidobacteria and, less frequently, streptococci, enterococci, *Bacillus* species, or yeast. Beneficial effects of probiotics include competitive exclusion of pathogens, normalization of perturbed microbiota, enhancement of intestinal barrier function, and differentiation and stimulation of systemic or mucosal immune responses (2, 3). Immunoregulatory probiotics, which are characterized by the ability to induce predominant IL-10 production, can promote the development of Treg cells and control inflammatory responses, resulting in a decrease in allergy, Inflammatory Bowel Disease, and autoimmune diseases (4). Although some of the above-mentioned effects might be widespread among common probiotic genera, others are species- or strain-specific (1).

Probiotic preparations for human consumption are marketed as medicinal products or foods (food supplements and fermented or novel foods) with new formulations being constantly introduced in the market. Conventional probiotic products contain mono-to-mixed cultures of microorganisms or bacterial spores and are marketed as oral pills, capsules, powder sachets, granulates, or suspensions.

The ESPGHAN Working Group for Probiotics and Prebiotics (5) recently indicated that orally administered probiotic formulations have to contain a sufficient number of living microorganisms by the end of shelf-life and to be contamination free. Investigations on the compositional quality of commercial human probiotic supplements available in Europe (6–10), South Africa (11, 12), Taiwan (13), India and Pakistan (14), and the USA (15) often indicated that their content does not correspond to the label information in terms of identity, viability, number of microorganisms, and purity. In the clinical context, the administration of probiotic products that do not comply quality requirements most likely leads to reduced/absent efficacy of the preparation and represents a potential infective risk for patients, if pathogens or opportunistic pathogens are present.

Desirable properties for oral probiotics are (i) to survive in the acidic environment they encounter during gastric transit and (ii) to be active, vital, and able to multiply in the intestine (5). Therefore, acid and bile resistance and, possibly, multiplication in the presence of bile are characteristics used to select potential probiotic strains.

To gain information on the gastric tolerance and bile resistance of probiotics, many *in vitro* studies have been performed. The results obtained demonstrated heterogeneous behavior depending on the species and strains analyzed (16–20). Therefore, knowledge of these functional properties for probiotic formulations appears essential in driving the choice of the clinicians between different products.

Italy has a long tradition of using beneficial bacteria to support the balance of intestinal flora. In 2013, the Italian Ministry of Health issued new guidelines on probiotics in food supplements, which provide indications that these organisms must be traditionally used for supplementation of the intestinal microflora in humans, be safe for human consumption, and be administered in a sufficient amount (1 × 10^9^ CFU per day) to be active and vital in the intestine (21).

In this study, we characterized the top 10 probiotic formulations commercialized in Italy, in terms of enumeration and identification of the contained organisms and assessment of their resistance to simulated gastric and intestinal juice. Our findings represent the first step required for predicting the effect of the analyzed products on clinical outcomes.

## Materials and Methods

### Probiotic Formulations

The formulations analyzed in this study are reported in Table 1. In the table, products were ordered on the basis of the most selling formulations of each of the top 10 umbrella brands in the Italian probiotic market sold in pharmacies in the last 12 months—IMS Sell-out value, MAT Mar-16. All formulations were purchased in pharmacies by the investigators and investigated before the expiration date.

**Table 1:** Probiotic products used in the study.

Product	Batch	Expiration date
Enterogermina 2mld vials	1739	03/2018
Enterolactis Plus capsules	1486	05/2018
Lactoflorene Plus bottles	1396	05/2018
Reuflor drops	6DSA026	03/2018
Codex capsules	1609	05/2018
Prolife bottles	260	05/2018
Dicoflor drops	F0533	09/2017
Enterelle capsules	1116	11/2017
Yovis sachets	EA160025	02/2018
VSL3 sachets	606035	06/2018

### Microbial Identification and Enumeration

Capsules and lyophilized preparations were dissolved in sterile water immediately before the analyses were performed. Formulations claimed to contain spore-forming microorganisms were divided into two aliquots. One aliquot was thermally treated at 80°C for 15 min prior to plating. Thermally treated and untreated suspensions were serially diluted in PBS and seeded (100 µL per plate) on trypticase soy agar with 5% horse blood (TSH, bioMérieux, Marcy l’Etoile, France). All formulations were serially diluted in PBS and plated on different media to selectively differentiate the contained species. Aliquots of the diluted products were seeded on TSH for the isolation of *Bacillus* spp., *Enterococcus* spp., and *Streptococcus* spp., on De Man, Rogosa & Sharpe agar (MRS, Oxoid, Thermo Fisher Scientific, UK) for *Lactobacillus* spp., and on *Bifidobacterium* selective medium (BSM, Sigma-Aldrich, Saint Louis, USA) for *Bifidobacterium* spp. (22). Plating was performed in triplicate and the experiments were repeated three times in separate days. TSH plates were incubated at 37°C in aerobic atmosphere for 48 h, MRS plates at 35°C in 5% CO_2_ for 72 h, and BSM plates at 37°C in anaerobic atmosphere for up to 72 h. The number of CFU was determined and representative colonies subjected to identification by biochemical analysis using the Vitek 2 system (bioMérieux, Marcy l’Etoile, France) with the GP card for *Enterococcus* spp. and *Streptococcus* spp. and the BCL card for *Bacillus* species. Bacteria from single colonies were used for MALDI-TOF mass spectrometry (MS) analysis with Flex Control TM 1.1 (Bruker Daltonics, Bremen, Germany) and spectra were analyzed by the MALDI Biotyper 3.0 (BDAL, Bruker Daltonics, Bremen, Germany).

#### MALDI-TOF MS Analysis

The isolates were tested in duplicate. A colony was directly spotted on the MALDI plate, treated with 1 µL of ethanol, 1 µL of formic acid, and 1 µL of acetonitrile and then overlaid with 1 µL of saturated α-cyano-4-hydroxycinnamic acid and air-dried. The loaded plate was then placed in the instrument according to the manufacturer’s instructions. The mass spectra were acquired within 10 min. The spectra were imported into the integrated MALDI Biotyper software (version 3.0) and analyzed by standard pattern matching with a default setting. A score ≥2.00 indicated identification at the species level, a score from 1.99 to 1.70 indicated identification at the genus level, whereas any score <1.70 meant no significant similarity of the obtained spectrum with any database entry.

#### Preparation of the Inocula for Viability Assays

Inocula were prepared as follows. Enterogermina, Reuflor, and Dicoflor suspensions were used as such. The lyophilized microorganisms constituting Lactoflorene Plus and Prolife were suspended in the liquid contained in the provided bottle, as recommended by the manufacturer’s instructions. The powder contained in one capsule of Enterolactis Plus, Codex and Enterelle was dissolved in 10 mL of sterile water. The powder contained in Yovis and VSL3 sachets was dissolved in 50 mL of sterile water.

### Microbial Viability in Simulated Gastric Juice

Two different artificial gastric juices were used. The first was a solution of 0.07 N hydrochloric acid with pH 1.5 at 37°C as specified by the American Society of Testing Materials (23). The second consisted of 0.03 M sodium chloride, 0.084 M hydrochloric acid, and 0.32% (w/v) pepsin with pH 1.4 at 37°C as recommended by the U.S. Pharmacopeia (USP) (24). Aliquots (100 µL) of each inoculum suspension or five drops (200 µL) of Reuflor and Dicoflor were inoculated in 5 mL of the ASTM or USP simulated gastric juice (25) and incubated at 37°C for 0, 30, 60, and 120 min. At each time point, 100-µL aliquots of the suspensions were serially diluted and seeded on TSH, MRS, and BSM. Plating was performed in triplicate and plates incubated in the conditions reported above. The number of CFU was determined and the CFU/unit dose of each product extrapolated. Microbial survival at the end of the treatment was calculated as follows: % survival = logCFU of viable cells survived/logCFU of initial viable cells inoculated × 100 (26). Three experimental replicates were performed.

### Microbial Viability in Simulated Intestinal Fluid

Simulated intestinal fluid was prepared by dissolving 0.3% w/v Oxgall bile salts (Sigma-Aldrich) and 0.1% w/v pancreatin (Sigma-Aldrich) in sterile saline solution (0.85% NaCl) and adjusting to pH 8.0 (27). Aliquots (100 µL) of each inoculum suspension or five drops (200 µL) of Reuflor and Dicoflor were inoculated in 5 mL of simulated intestinal fluid and incubated at 37°C for 0, 30, 60, 120, 240, and 360 min. At each time point, aliquots (100 µL) of the microbial suspensions were serially diluted and seeded on TSH, MRS, and BSM. Plating was performed in triplicate and plates incubated in the conditions reported above. The number of CFU was determined and the CFU/unit dose of each product extrapolated. The experiments were repeated three times in separate days.

### Statistical Analysis

Data were expressed as the mean ± SD. Statistical analysis was performed by applying the one-way ANOVA analysis-repeated measures with Dunnett’s correction. A *P*-value <0.05 was considered significant.

## Results

### Enumeration and Identification of the Organisms Contained in Probiotic Formulations

Considering the importance of compositional quality for commercial probiotic products, particularly for medicinal products, we analyzed the formulations reported in Table 1 in terms of enumeration and identification of the contained organisms. Table 2 reports the labeled number of cells, the total counts (total CFU), and the counts of spores (CFU from spores only) obtained for a unit dose (one vial, one capsule, one bottle, one sachet or five drops) of each product. Total CFU were concordant with the labeled number of cells for Enterogermina, Codex, Prolife, and Dicoflor. Lactoflorene Plus produced a lower CFU number per unit dose than that declared by the manufacturer. Total CFU originating from Enterolactis Plus, Reuflor, Enterelle, Yovis, and VSL3 were 1–3 log higher than those labeled. The amount of spores contained in Enterogermina, Lactoflorene Plus, and Prolife was concordant with the labeled amount of *B. clausii* spores and *B. coagulans* (2 × 10^9^, 2 × 10^7^, ≥10^9^, respectively).

**Table 2:** Enumeration of the organisms contained in a unit dose of each probiotic formulation.

Formulation	Dose	Labeled cell no.	Total CFU	CFU from spores only
Enterogermina	1 vial	2 × 10^9^	1.15 ± 0.50 × 10^9^	1.65 ± 0.71 × 10^9^
Enterolactis Plus	1 capsule	2.4 × 10^10^	2.71 ± 0.30 × 10^12^	
Lactoflorene Plus	1 bottle	2 × 10^9^	6.02 ± 5.73 × 10^7^	1.35 ± 1.50 × 10^7^
Reuflor	5 drops	1 × 10^9^	8.72 ± 1.53 × 10^11^	
Codex	1 capsule	5 × 10^9^	2.68 ± 2.4 × 10^9^	
Prolife	1 bottle	1.25 × 10^11^	2.16 ± 0.36 × 10^11^	3.51 ± 1.49 × 10^10^
Dicoflor	5 drops	5 × 10^9^	9.65 ± 1.95 × 10^9^	
Enterelle	1 capsule	3 × 10^9^	5.74 ± 0.99 × 10^10^	
Yovis	1 sachet	2.97 × 10^11^	3.51 ± 3.13 × 10^12^	
VSL3	1 sachet	4.5 × 10^11^	4.53 ± 0.47 × 10^13^	

All morphologically different colonies isolated from each product were subjected to identification by MALDI-TOF MS. Biochemical identification was also applied to *Bacillus, Streptococcus*, and *Enterococcus* spp. Table 3 reports the results of all the identification procedures. Concordant results were obtained by MALDI-TOF MS and biochemical testing. All the identified species corresponded to those labeled by the manufacturers. No contaminant microorganism was found in any product. *Lactobacillus delbrueckii* subsp. *bulgaricus*, which is declared to be present in Yovis and VSL3, was identified only at the genus level. Possibly due to the difficulty to recognize the colonies produced by different *Bifidobacterium* species and to the genus complexity (28), we were not able to identify the three *Bifidobacterium* species labeled on Yovis and VSL3.

**Table 3:** Identification of the microorganisms contained in each probiotic formulation.

Formulation	Labeled organisms	Biochemical identification	MALDI-TOF MS identification
Enterogermina	*Bacillus clausii* spores	*B. clausii*	*B. clausii*

EnterolactisPlus	*Lactobacillus paracasei* CNCM I-1572		*L. paracasei*

LactoflorenePlus	*L. acidophilus* LA-5^®^ *L. paracasei* CRL 431^®^ *Bifidobacterium* BB-12^®^ *B. coagulans* BC513	*B. coagulans*	*L. acidophilus* *L. paracasei* *B. animalis* subsp. *lactis* *B. coagulans*

Reuflor	*L. reuteri* DSM 17938		*L. reuteri*

Codex	*Saccharomyces boulardii*		*S. cerevisiae*

Prolife	*B. coagulans* MTCC 5260 *Bifidobacterium lactis* HN019 *Streptococcus thermophilus* St-21 *L. acidophilus* La-14 *L. plantarum* Lp-115 *L. brevis* Lbr-35 *L. rhamnosus* HN001 *L. casei* R215 *L. gasseri* Lg-36 *L. helveticus* R0052	*B. coagulans* *S. salivarius* subsp. *thermophilus*	*B. coagulans* *B. animalis* subsp. *lactis* *S. salivarius* *L. acidophilus* *L. plantarum* *L. brevis* *L. rhamnosus* *L. casei* *L. gasseri* *L. helveticus*

Dicoflor	*L. rhamnosus* GG		*L. rhamnosus*

Enterelle	*S. cerevisiae* sub*. boulardii* MTCC-5375 *Enterococcus faecium* UBEF-41 *L. acidophilus* LA 14	*E. faecium*	*S. cerevisiae* *E. faecium* *L. acidophilus*

Yovis	*S. salivarius* subsp. *thermophilus* *B. breve* *B. infantis* *B. longum* *L. acidophilus* *L. plantarum* *L. casei* *L. delbrueckii* subsp. *bulgaricus* *S. faecium*	*S. salivarius* subsp. *thermophilus* *E. faecium*	*S. salivarius* *Bifidobacterium* spp. *L. acidophilus* *L. plantarum* *L. casei* *L*. spp. *E. faecium*

VSL3	*S. thermophilus* BT01 *B. breve* BB02 *B. longum* BL03 *B. infantis* BI04 *L. acidophilus* BA05 *L. plantarum* BP06 *L. paracasei* BP07 *L. delbrueckii* subsp. *bulgaricus* BD08	*S. salivarius* subsp. *thermophilus*	*S. salivarius* *Bifidobacterium* spp. *L. acidophilus* *L. plantarum* *L. paracasei* *Lactobacillus* spp.

### Microbial Survival in Simulated Gastric Juice

**Figure 1:**
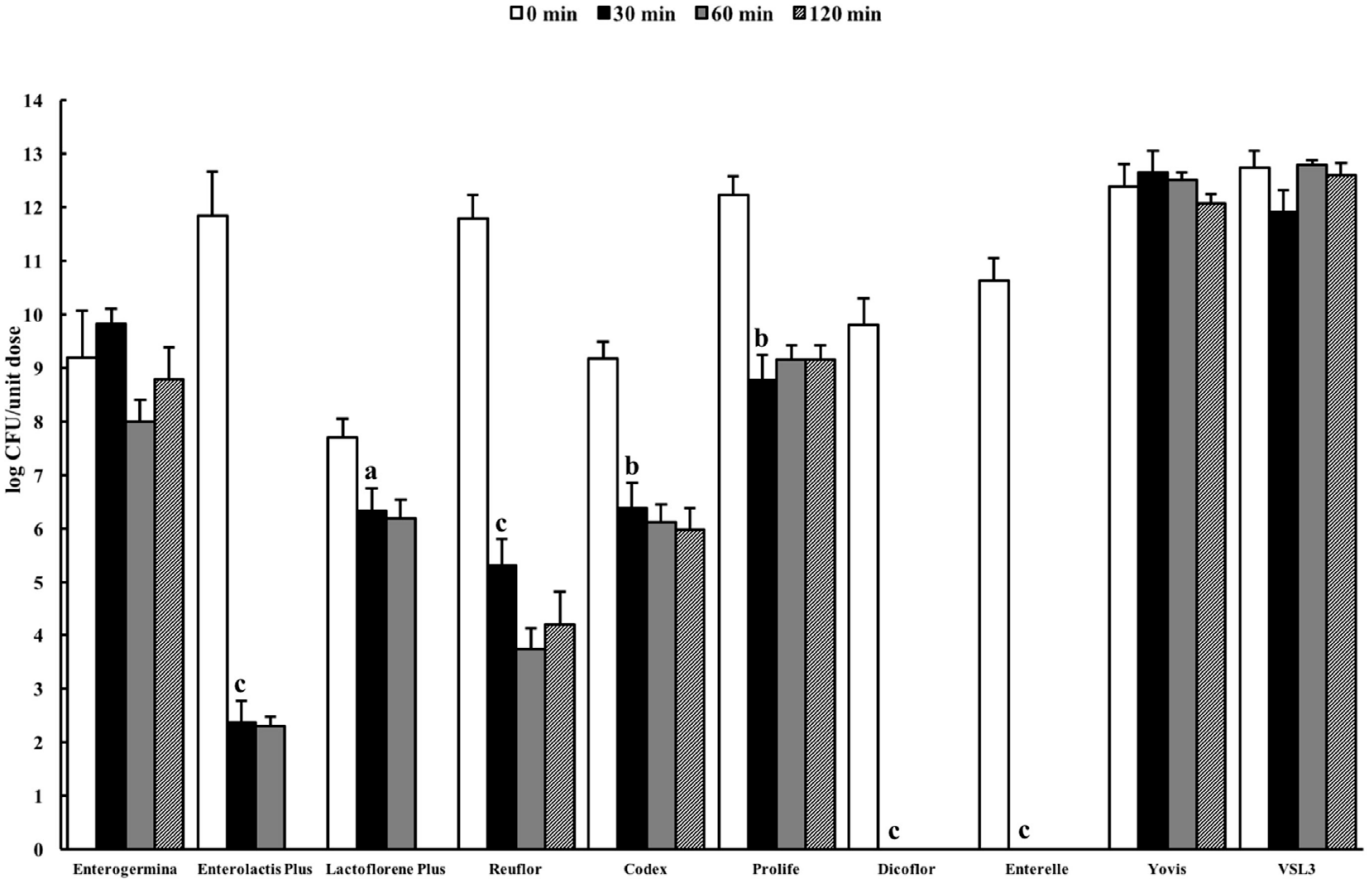
Viability of probiotic formulations in the ASTM-simulated gastric fluid. Microbial counts were carried out at 0, 30, 60, and 120 min and expressed as log CFU/unit dose of each product. ^a^*P* < 0.05, ^b^*P* < 0.01, and ^c^*P* < 0.001.

Resistance to stomach pH is important in predicting the potential survival of probiotics in the gastrointestinal conditions. Gastric pH varies from 1.5 to 3.5 in the human stomach lumen (29). In this study, we decided to evaluate the behavior of the selected probiotic formulations in two standard simulated gastric fluids, both characterized by a low pH (1.4–1.5), containing (USP) or not (ASTM) pepsin, in order to mimic an extremely harsh gastric environment. Figure 1 shows the results of the total counts obtained at 0 min and after 30-, 60-, and 120-min incubation for a unit dose of each formulation in the ASTM artificial gastric juice. The majority of products (Enterolactis Plus, Lactoflorene Plus, Reuflor, Codex, Prolife, Dicoflor, Enterelle) showed a significant reduction (*P* < 0.05) in the number of viable cells already after 30 min of incubation in the artificial gastric juice. At this incubation time, no residual CFU were found for Dicoflor and Enterelle. On the other hand, the bacteria contained in Enterogermina, Yovis, and VSL3 were found to be able to tolerate the acidic condition of the juice as long as 120 min. At this time, the% survival of the organisms contained in these formulations was 96, 97, and 99%, respectively. At the end of the incubation in the artificial gastric juice, a 36, 65, and 75% survival was registered for Reuflor, Codex, and Prolife, respectively. No residual alive organisms were found in Enterolactis Plus and Lactoflorene Plus after 120 min of incubation.

**Figure 2:**
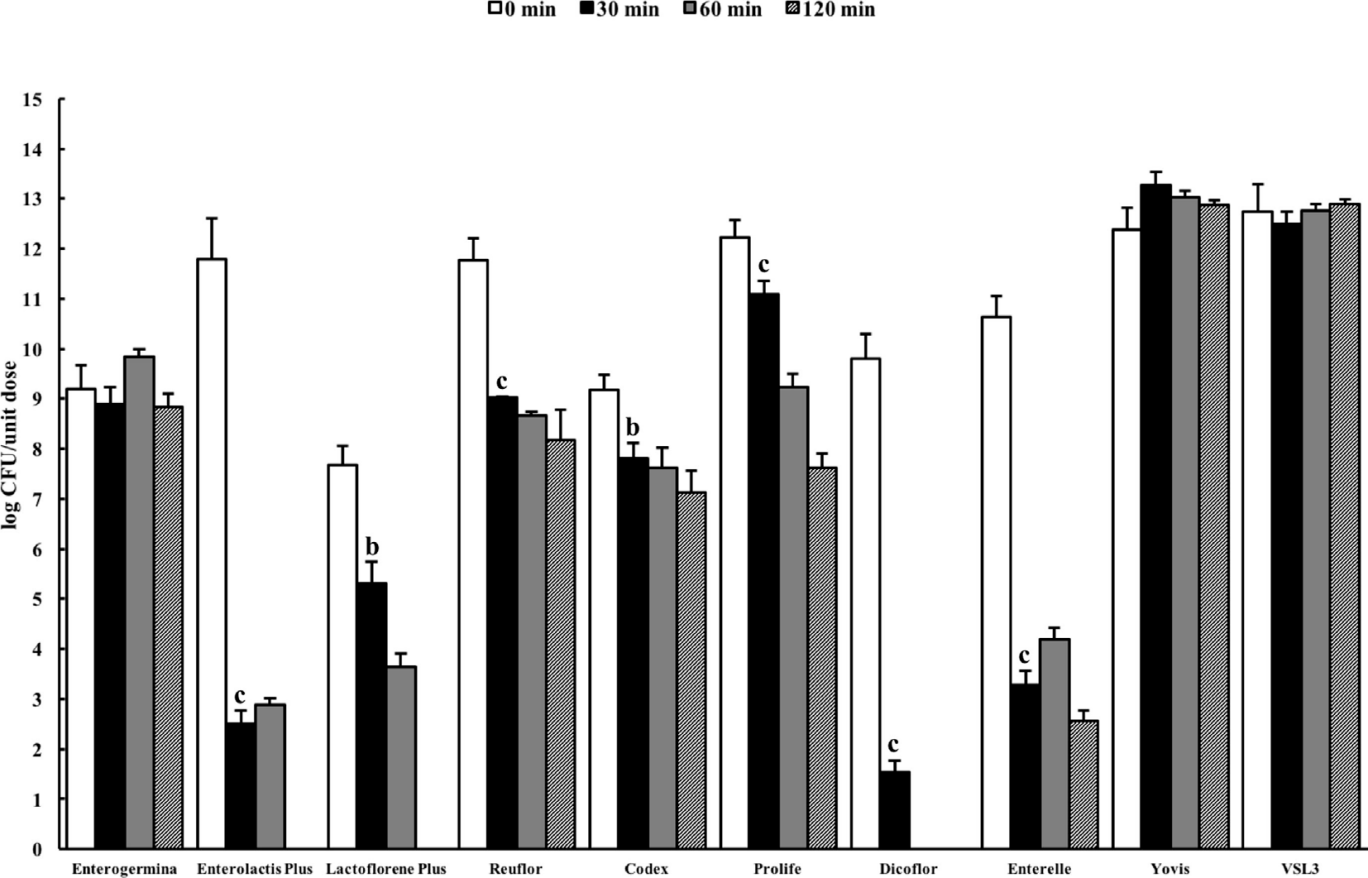
Viability of probiotic formulations in the U.S. Pharmacopeia simulated gastric fluid. Microbial counts were carried out at 0, 30, 60, and 120 min and expressed as log CFU/unit dose of each product. ^a^*P* < 0.05, ^b^*P* < 0.01, and ^c^*P* < 0.001.

Figure 2 shows the results of the total counts obtained at 0 min and after 30-, 60-, and 120-min incubation in the USP artificial gastric juice. Similar to the results obtained in the ASTM juice, Enterolactis Plus, Lactoflorene Plus, Reuflor, Codex, Prolife, Dicoflor, and Enterelle showed a significant reduction (*P* < 0.01) in the number of viable cells after 30 or 60 min of incubation in the artificial gastric juice. At the end of the incubation in the artificial gastric juice, no residual colonies were found for Enterolactis Plus, Lactoflorene Plus, and Dicoflor, a 24% survival was obtained for Enterelle, while Reuflor, Codex, and Prolife showed a 69, 78, and 62% survival, respectively. Vitality of the microorganisms present in Enterogermina, Yovis, and VSL3 was not affected by the incubation in the juice as long as 120 min.

### Microbial Behavior in Simulated Intestinal Juice

**Figure 3:**
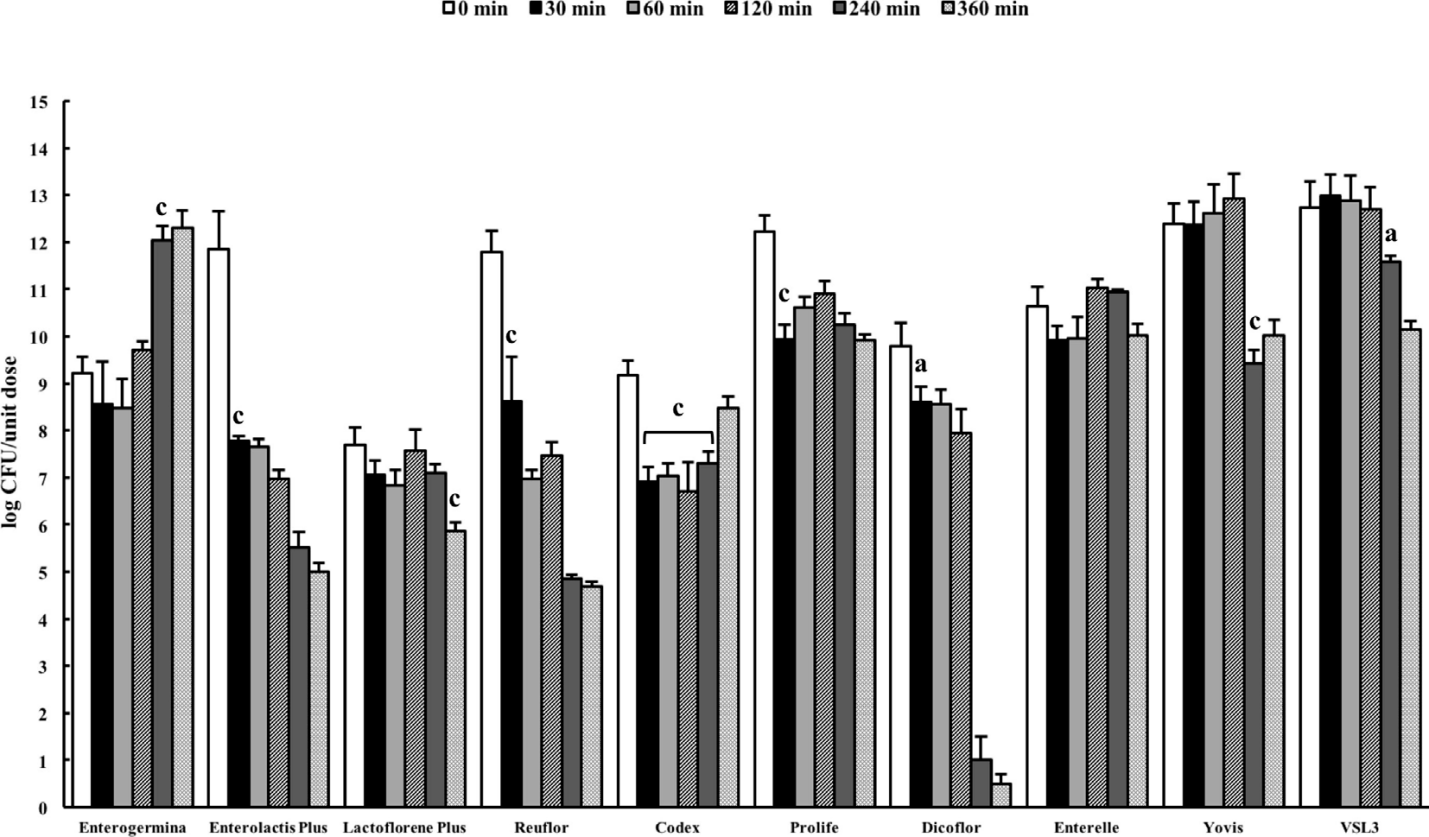
Behavior of probiotic formulations in simulated intestinal juice. Microbial counts were carried out at 0, 30, 60, 120, 240, and 360 min and expressed as log CFU/unit dose of each product. ^a^*P* < 0.05, ^b^*P* < 0.01, and ^c^*P* < 0.001.

The pancreatin–bile salt solution (pH 8.0) aims at simulating the conditions of the intestine (27). The probiotic formulations were dissolved in the solution and the number of viable organisms present in the suspension was quantified by plating at 0 min and after incubation for 30, 60, 120, 240, and 360 min. A very different behavior was observed among the probiotic formulations (Figure 3). A significant reduction in cell viability was recorded for Enterolactis Plus, Reuflor, Prolife, and Dicoflor starting from 30 min of incubation, for Yovis and VSL3 starting from 240 min, and for Lactoflorene Plus at 360 min. No variation in cell viability was observed for Enterelle. Interestingly, the bacteria present in Enterogermina were found able to replicate in the juice, with a significant increase in their number being recorded starting from 240 min of incubation. Following an initial decrease at 30 min, the *S. cerevisiae* strain contained in Codex was able to replicate in the juice reaching at 360 min a number of cells comparable with 0 min (Figure 3).

## Discussion

The effectiveness of a probiotic product is the result of its microbial quality, resistance to harsh gastric environment, and exertion of functional properties, such as anti-oxidant, antimicrobial, and immunomodulatory abilities (2, 30–32).

The number of viable cells contained in a probiotic formulation is one of the qualifications that the Food and Agriculture Organization (FAO) and the World Health Organization (WHO) document (33) have recommended. According to the Italian Ministry of Health guidelines on food supplements based on probiotics the daily uptake of a probiotic should be at least 1 × 10^9^ CFU. Lower numbers of viable microorganisms could preclude an effective health benefit. This investigation hence intends to clarify whether probiotics on the Italian market comply with these general quality requirements. Since routine industrial controls for probiotic products are mainly carried out by traditional plate count techniques (34), herein plate counting on non-selective and selective media was chosen as the method for enumerating the microorganisms contained in the formulations. The results obtained indicate that, with the exception of the examined batch of Lactoflorene Plus, all the other formulations contain more than 1 × 10^9^ CFU per unit dose. The *Bacillus* species declared to be contained in Enterogermina, Lactoflorene Plus and Prolife were all represented by heat-resistant bacterial forms (spores).

MALDI-TOF MS has successfully been applied for the identification of bacteria present in probiotics (35). In this study, MALDI-TOF MS was able to correctly identify all the organisms contained in the analyzed probiotic formulations, with the exception of *L. delbrueckii* subsp. *bulgaricus*. This species was previously shown to be identifiable by the MALDI Biotyper 3.0 software (36). In our case, we can only hypothesize that the low score values obtained for this bacterium are due to the limited number of strains of this species included in the MALDI-TOF database (*n* = 1). In fact, commercial databases are mostly designed for the identification of species that are encountered at higher frequency in clinical practice.

By using both MALDI-TOF MS and Vitek2, all the formulations were shown to contain the labeled species. In Lactoflorene Plus, *Bifidobacterium* BB-12 resulted as *B. animalis* subsp. *lactis*, as reported in the literature for such a strain (37). In Prolife, the labeled *B. lactis* HN019 resulted to be *B. animalis* subsp. *lactis*, as indicated by the current nomenclature. *S. faecium* labeled on Yovis was correctly identified as *E. faecium*, and *S. thermophilus* St-21 and *S. thermophilus* BT01 (Prolife and VSL3), as *S. salivarius* subsp. *thermophilus*, as recommended by updated nomenclature.

Oral probiotics must be capable of surviving passage through the gastric environment. Resistance to gastric harsh condition has been evaluated in a variety of different conditions, in terms of composition of the juice, pH and times, using both simulated gastric juice or animal and human fluids (38). Although the use of artificial fluids does not consider the influence of dietary and nonacid constituents of gastric secretions, it has the benefit of not being restricted by the availability of animal-derived material. Moreover, it provides more controllable and homogenous experimental conditions to compare the effect of acidity on different probiotic products.

The *B. clausii* spore suspension contained in Enterogermina [strains O/C, SIN, N/R, T; Ref. (39)] tolerated the acidic conditions of both ASTM and USP gastric juices well for 120 min. This result could be expected, since bacterial spores are well known for their ability to survive in extreme environments (pH, temperature, salinity, etc.). The finding that the *B. coagulans* spores contained in Lactoflorene Plus (1.35 ± 1.50 × 10^7^) underwent progressive inactivation in both juices (Figure 2) can be explained with the diverse susceptibility of spores derived from different bacteria toward acids. *B. coagulans* spores have, indeed, already been reported to be gradually inactivated at pH 1.5 (40).

*Lactobacillus* species are considered intrinsically resistant to acids, but tolerance to gastric environments is a species- and strain-specific property (38). Our results on formulations only containing one *Lactobacillus* strain are in line with this characteristic of the genus. Polymicrobial formulations, such as Yovis and VSL3, displayed good resistance to the acidic conditions of both juices. Although we did not investigate the outcome of each of the species contained in such formulations, it can be speculated that their complexity potentially favor resistance and adaptability of these microbial consortia to different environmental stresses.

Upon reaching the intestine, probiotics encounter alkaline conditions and are exposed to the effect of bile salts. In general, Gram-positive bacteria are more sensitive to the deleterious effects of bile than Gram-negative bacteria, although in a strain-specific manner (41). The use of artificial intestinal fluids is a good model for analyzing the survival of microorganism during transit in the gut (27). In this study, seven of the considered formulations (Enterolactis Plus, Reuflor, Prolife, Dicoflor, Yovis, VSL3, Lactoflorene Plus) underwent a significant reduction in the number of viable organisms following incubation in the intestinal juice. Nevertheless, the time required for microbial inactivation was very different from one formulation to another, with Lactoflorene Plus, Yovis, and VSL3 formulations being the most resistant to the intestinal juice. Interesting behavior was observed for Codex, a monomicrobial preparation of *S. cerevisiae*. In fact, after an initial 2-log decrease in the number of viable cells, the strain was able to multiply almost regaining the initial *t*_0_ number of cells. This can result from a precocious killing of a certain amount of yeast cell followed by a tardive multiplication of the residual population, which is in line with *S. cerevisiae* doubling time (90–140 min). Peculiar behavior was also observed for Enterogermina. *B. clausii* was found to be able to replicate in the intestinal juice, with a significant increase in the number of cells starting from 240 min of incubation. This finding indicates that the *B. clausii* spores contained in Enterogermina can germinate and actively multiply in the intestinal fluid. *B. clausii* adaptability to the alkaline environment of the juice might be the consequence of the alkaliphilic nature of this *Bacillus* species (42). At present, we are unable to explain the resistance of *B. clausii* to bile salts. Nevertheless, this finding correlates with previous data from an *in vivo* study on human volunteers indicating that Enterogermina *B. clausii* (strains O/C, SIN, N/R, T) multiplies in the human intestine (39).

In conclusion, the results of this study indicate high quality of the examined probiotic preparations and highlight their different behavior in the presence of acid and bile. Apart from a minor drawback in the identification of *L. delbrueckii* subsp. *bulgaricus*, MALDI-TOF MS demonstrated an excellent applicability for the identification of the microorganisms constituting the preparations. The analysis of microbial survival in extreme acid conditions aimed at mimicking the worst environment these microorganisms may encounter during transit in the stomach lumen. Our results indicate that amount of microorganisms contained in Enterogermina, Yovis, and VSL3 is not reduced in these conditions for up to 2 h. The behavior the formulations exhibited in the presence of pancreatin and bile salts at pH 8 was very different. Enterogermina *B. clausii* was the only one able to multiply significantly above the initial amount and Codex *S. boulardii* to replicate up to the starting amount after an initial decline in the artificial intestinal juice.

The integration of these artificial *in vitro* studies with functional and immunological investigations appears essential for selecting strains or mixtures that hold the potential for probiotic application.

## Author Contributions

AV, FC, and DM wrote and critiqued the experimental protocol and performed the experimental work. EG conceived the research idea, analyzed the data, and wrote the manuscript. AL and SS critiqued the experimental protocol and edited the manuscript.

## Conflict of Interest Statement

EG has been a lecturer for Sanofi S.p.A. AV, FC, DM, SS, and AL have no conflict of interest to declare. The reviewer LRL and the handling Editor declared their shared affiliation.
