# Corrigendum: Compositional quality and potential gastrointestinal behavior of probiotic products commercialized in Italy

**DOI:** 10.3389/fmed.2022.1091788

**Published:** 2023-01-27

**Authors:** Alessandra Vecchione, Francesco Celandroni, Diletta Mazzantini, Sonia Senesi, Antonella Lupetti, Emilia Ghelardi

**Affiliations:** ^1^Department of Translational Research and New Technologies in Medicine and Surgery, University of Pisa, Pisa, Italy; ^2^Department of Biology, University of Pisa, Pisa, Italy; ^3^Research Center Nutraceuticals and Food for Health-Nutrafood, University of Pisa, Pisa, Italy

**Keywords:** probiotics, microbial identification, MALDI-TOF, gastric juice, intestinal fluid, acid resistance, bile tolerance

In the published article, there was an error in Table 2 as published. The total CFU of VSL3 in Table 2 was displayed as “4.53 ± 0.47 × 10^13^.” The correct total CFU of VSL3 is “4.53 ± 0.47 × 10^12^.” The total CFU of Codex in Table 2 was displayed as “2.68 ± 2.4 × 10^9^.” The correct total CFU of Codex is “2.68 ± 2.40 × 10^9^.” The corrected Table 2 and its caption appear below.

**Table 2:** Enumeration of the organisms contained in a unit dose of each probiotic formulation.

**Formulation**	**Dose**	**Labeled cell no**.	**Total CFU**	**CFU from spores only**
Enterogermina	1 vial	2 × 10^9^	1.15 ± 0.50 × 10^9^	1.65 ± 0.71 × 10^9^
Enterolactis Plus	1 capsule	2.4 × 10^10^	2.71 ± 0.30 × 10^12^	
Lactoflorene Plus	1 bottle	2 × 10^9^	6.02 ± 5.73 × 10^7^	1.35 ± 1.50 × 10^7^
Reuflor	5 drops	1 × 10^9^	8.72 ± 1.53 × 10^11^	
Codex	1 capsule	5 × 10^9^	2.68 ± 2.40 × 10^9^	
Prolife	1 bottle	1.25 × 10^11^	2.16 ± 0.36 × 10^11^	3.51 ± 1.49 × 10^10^
Dicoflor	5 drops	5 × 10^9^	9.65 ± 1.95 × 10^9^	
Enterelle	1 capsule	3 × 10^9^	5.74 ± 0.99 × 10^10^	
Yovis	1 sachet	2.97 × 10^11^	3.51 ± 3.13 × 10^12^	
VSL3	1 sachet	4.5 × 10^11^	4.53 ± 0.47 × 10^12^	

